# Supplementary material for: Microbiota Transplantation in Individuals with Type 2 Diabetes and a High Degree of Insulin Resistance
Source: Nutrients. 2024 Oct 15;16(20):3491. doi: 10.3390/nu16203491 (PMC11510444; doi:10.3390/nu16203491)
Supplement: Supplementary file 1 [file nutrients-16-03491-s001.zip › nutrients-3238350-supplementary.pdf]

Gut microbiota analysis

Fecal samples were collected and immediately stored at – 80°C until analysis. DNA extraction from stools was performed with the QIAamp DNA stool Mini kit (Qiagen, Hilden, Germany) according to the manufacturer’s instructions. DNA concentrations were determined by absorbance at 260 nm (A260), and purity by determining the A260/A280 ratio with a Nanodrop spectrophotometer (Nanodrop Technologies). Ribosomal 16S rRNA gene sequences were amplified from DNA using the 16S Metagenomics Kit (Thermo Fisher Scientific, Italy). The kit includes two first sets that selectively amplify the corresponding hypervariable regions of the 16S region in bacteria: set V2–4–8, and set V3–6, 7–9. Libraries were created using the Ion Plus Fragment Library Kit (Thermo Fisher Scientific). Barcodes were added to each sample using the Ion Xpress Barcode Adapters kit (Thermo Fisher Scientific). Emulsion PCR and sequencing of the amplicon libraries were performed on an Ion 530 chip (Ion 530TM Chip Kit) using the Ion Torrent S5TM system and the Ion 510/520TM/530TM Kit-Chef (Thermo Fisher Scientific) according to manufacturer ́s instructions. After sequencing, the individual sequence readings were filtered using Ion Reporter Software V4.0 to remove low quality and polyclonal sequences. Profiling was performed within the tool Ion Reporter (Ion Reporter Software 5.12, Thermofisher), clustering with the reference base Greengenes version 13_5 at 99% of identity, and the curated MicroSEQ® 16S Reference Library V2013.1 at the species level. Feature tables were used to look for shared species or not with Venn diagrams.
